# Research progress in the treatment of non-scarring alopecia: mechanism and treatment

**DOI:** 10.3389/fphar.2025.1544068

**Published:** 2025-05-23

**Authors:** Rui-Xian Guo, Yong-Kang Zhao, Ke-Jian Hu, Kun-Mu Jia, Wei Shi, Yan-Xiao Yi, Hai-Ying Gong, Jia-Bo Wang, Yuan Gao

**Affiliations:** ^1^ School of Traditional Chinese Medicine, Capital Medical University, Beijing, China; ^2^ College of Pharmacy, Chengdu University of Traditional Chinese Medicine, Chengdu, China; ^3^ Beijing Obstetrics and Gynecology Hospital, Capital Medical University, Beijing Maternal and Child Healthcare Hospital, Beijing, China; ^4^ State Key Laboratory for Quality Ensurance and Sustainable Use of Dao-di Herbs, Beijing, China

**Keywords:** non-cicatricial alopecia, androgenetic alopecia, therapeutic drugs, Chinese medicine, novel treatments

## Abstract

**Introduction:**

Non-scarring alopecia, encompassing androgenetic alopecia (AGA), alopecia areata (AA), and telogen effluvium (TE), is a common skin condition that significantly impacts both the physical and psychological wellbeing of affected individuals. This review aims to delve into the multifactorial etiology of non-scarring alopecia and to critically assess the current and emerging treatment options available for management.

**Methods:**

The review methodology involved a comprehensive literature search in PubMed and Web of Science, focusing on the genetic, environmental, and psychological factors that contribute to the development of non-scarring alopecia. The search included studies published from 2004 to 2024, primarily in English, to incorporate both foundational and recent advancements in the field. The inclusion criteria encompassed clinical trials, meta-analyses, and high-quality observational studies investigating the pathogenesis and treatment of non-scarring alopecia. Case reports, editorials, and studies with insufficient methodological details were excluded. Additionally, this review evaluated FDA-approved treatments (e.g., minoxidil and finasteride) and emerging therapeutic agents, such as Janus kinase (JAK) inhibitors and natural remedies, with an emphasis on their mechanisms, efficacy, and safety profiles.

**Results:**

The findings from this review indicate that the currently available treatments, such as topical and oral minoxidil, oral finasteride, and Janus kinase (JAK) inhibitors, exhibit limited efficacy and are associated with adverse effects. In contrast, natural remedies have shown promise as alternative treatments, potentially offering more effective management with fewer side effects.

**Discussion:**

The conclusion drawn from this review is that there is a significant potential for natural products and innovative drugs to provide effective treatment options for non-scarring alopecia. However, the assumption that natural remedies universally have fewer side effects than conventional treatments requires careful consideration, as their safety profiles vary. This underscores the need for further research and development in this area to improve patient outcomes and quality of life.

## 1 Introduction

Alopecia, a condition affecting millions, manifests in two primary forms: cicatricial and non-cicatricial, each with distinct characteristics and implications (Martinez-Lopez et al., 2020; Alessandrini et al., 2021). Cicatricial alopecia, often referred to as “scarring alopecia,” is marked by the permanent impairment of hair follicle integrity, supplanted by fibrous tissue, typically the consequence of inflammatory, traumatic, or infectious agents that lead to follicular destruction and irreversible hair loss (Templeton and Solomon, 1994). The classification of cicatricial alopecia is refined by the inflammatory processes and mechanisms of hair follicle demise, distinguishing between primary cicatricial alopecia (PCA) and secondary cicatricial alopecia (SCA). In a pivotal classification by the North American Hair Research Society in 2001, PCA was categorized into lymphocytic, neutrophilic, mixed, and nonspecific subtypes, based on the predominant inflammatory infiltrate identified through scalp biopsy (Olsen et al., 2003).

The diagnostic approach to cicatricial alopecia necessitates a comprehensive medical history and physical examination to ascertain the condition’s reversibility, with scalp biopsy serving as an essential diagnostic tool to identify the specific alopecia variant (Alessandrini et al., 2021; Phillips et al., 2017; Stefanato, 2010). Non-cicatricial alopecia, on the other hand, is influenced by a spectrum of factors including genetic predispositions, autoimmune conditions, hormonal imbalances, and nutritional deficiencies (Rushton, 2002). This category encompasses a range of conditions such as androgenic alopecia (AGA), alopecia areata (AA), trichotillomania, and various hair cycle disruptions, characterized by alterations in the hair cycle (HC) and diminished stem cell activity within the hair follicle (Owczarczyk-Saczonek et al., 2018).

The interplay between stress and alopecia has emerged as a significant area of research, with evidence suggesting a complex causal relationship (Ito, 2010; Peters et al., 2006). Psychological distress associated with alopecia can be exacerbated by factors such as headwear pressure and the emotional strain of social environments, with a notable prevalence of stress-related alopecia among younger demographics (Zhuang et al., 2021). Moreover, lifestyle factors, including irregular eating habits, excessive alcohol consumption, and smoking, have been identified as potential contributors to alopecia (Trüeb and Dias, 2018). Despite the widespread prevalence of alopecia, the existing treatment methods and their efficacy cannot fulfill the demand.

Thus far, the drugs approved by the US Food and Drug Administration (FDA) to treat alopecia are minoxidil, finasteride, ritlecitinib, baricitinib, and a device, low-level laser therapy (LLLT). Minoxidil, finasteride, and LLLT are mainly used in the treatment of AGA. Minoxidil can also be used to treat AA and chemotherapy-induced alopecia, but its effectiveness is generally limited to the early or middle stages of alopecia, showing little or no effect in the late stages (Adil and Godwin, 2017). Both minoxidil and finasteride can have some side effects. Minoxidil may cause itching and erythema, whereas finasteride may lead to sexual dysfunction, erectile dysfunction, and gynecomastia (Rossi et al., 2012). The JAK inhibitors, Ritlecitinib and Baricitinib, offer promising therapeutic avenues for severe alopecia areata, yet their side effects mandate vigilant monitoring and management.

The quest for novel therapeutics in alopecia is imperative, driven by the unmet clinical needs and the recognition of the multifactorial nature of this condition. Innovation in treatment strategies is essential to address the complexity of alopecia, with a focus on safety, efficacy, and patient-centered outcomes.

## 2 Common non-cicatricial alopecia and its mechanisms

**FIGURE 1:**
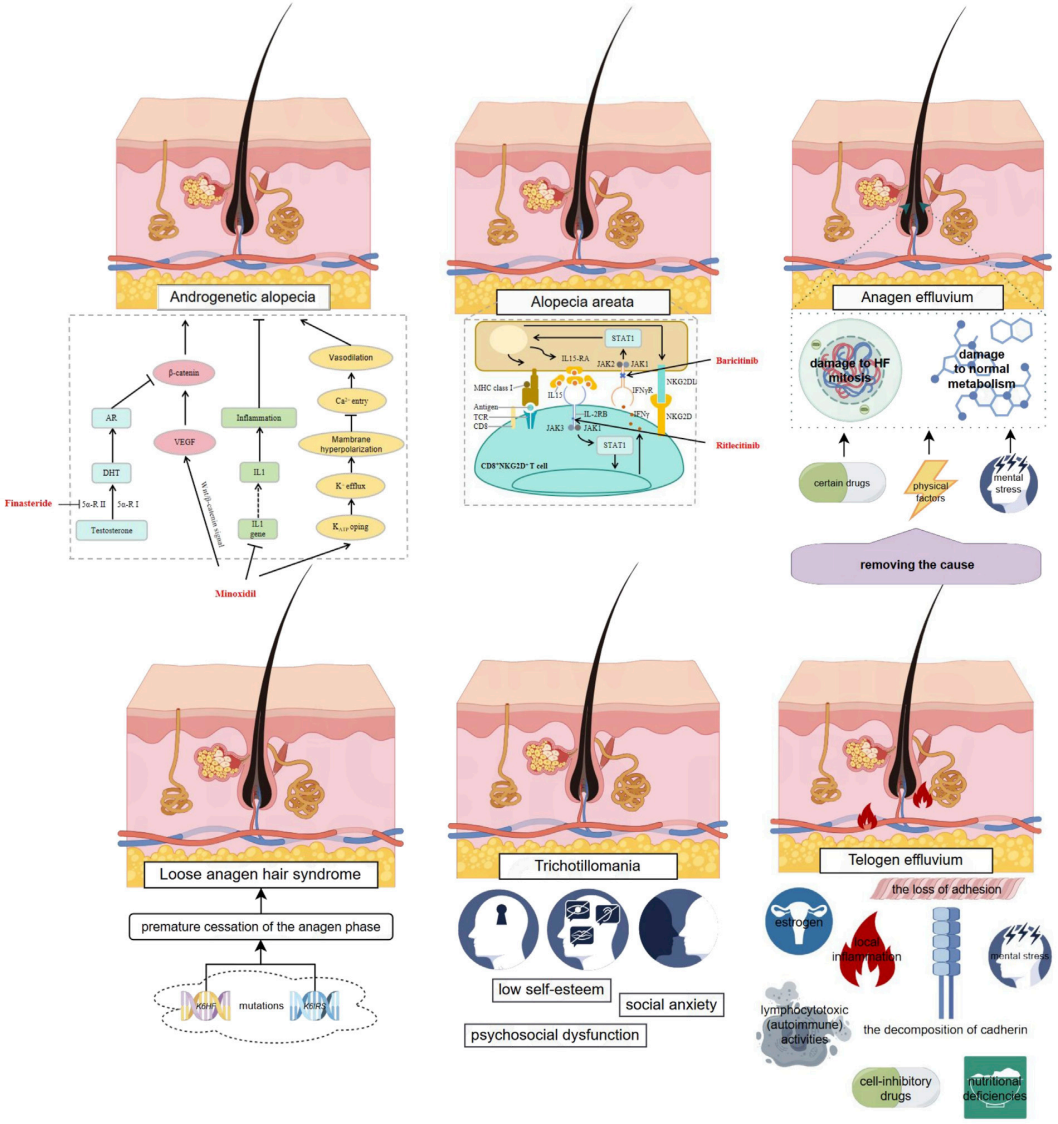
Complex Mechanisms of Non-cicatricial Alopecia and Existing Small Molecule Therapies. This figure provides an overview of various types of non-cicatricial alopecia, including androgenetic alopecia, anagen effluvium, alopecia areata, telogen effluvium, trichotillomania, and loose anagen hair syndrome, each characterized by distinct pathophysiological features. It illustrates the complex interplay of genetic, metabolic, and environmental factors contributing to hair loss. Existing approved small molecule therapies are highlighted in red, with arrows indicating their mechanisms of action as reported in current literature. The figure was created using Figdraw (www.figdraw.com).

To advance the scientific understanding and therapeutic intervention of hair loss, a meticulous examination of the contributing factors to non-cicatricial alopecia is imperative. Figure 1 delineates a detailed panorama of non-cicatricial alopecia, showcasing its various manifestations, including androgenetic alopecia, anagen effluvium, alopecia areata, telogen effluvium, trichotillomania, and loose anagen hair syndrome. Each subtype is distinguished by its own set of pathophysiological mechanisms, which are intricately interwoven. This visual dissection not only facilitates a deeper appreciation of the condition’s layered complexity but also accentuates the progressive strides in dermatological therapeutics. It serves as a scientific compendium that encapsulates the current state of knowledge and the evolving strategies in the management of non-cicatricial alopecia.

### 2.1 Androgenetic alopecia

Androgenetic alopecia (AGA), also referred to as seborrheic alopecia or premature baldness, is a prevalent form of non-cicatricial alopecia that affects individuals of both genders. Predominantly observed in the Caucasian population, AGA is estimated to impact approximately 80% of men and 40% of women under the age of 70, highlighting its widespread nature within this demographic (Gentile and Garcovich, 2019). The prevalence of AGA escalates with advancing age, post-puberty, reaching an astonishing 90% by the age of 80 among Caucasians. The condition bears a strong genetic predisposition, with paternal inheritance being more probable than maternal, underscoring the role of heredity in its development.

AGA is differentiated into two primary forms: male pattern baldness, known as MPHL, and its female counterpart, FPHL. The specific role that androgens play in FPHL is still under investigation, yet the term FPHL has become more widely recognized for its precision in characterizing the pattern of hair loss in women (Bertoli et al., 2020). The patterns of hair loss exhibit gender-specific differences. Men typically experience a receding hairline at the temples and thinning at the crown, while women see a more uniform thinning across the frontal to the posterior scalp without a receding hairline (Carmina et al., 2019).

A defining characteristic of AGA is the non-cicatricial and incremental reduction in the size of hair follicles, which shortens the growth phase and extends the resting phase, culminating in the emergence of finer, less pigmented hairs across the scalp (Katzer et al., 2019). Patients with AGA may present with symptoms such as reduced hair shaft thickness, changes in the density of hair coverage, lighter hair strands, and increased sebaceous gland activity marked by inflammation indicators like redness, itching, and discomfort (Kelly et al., 2016).

Clinical assessment of AGA severity is conducted through established grading systems. The Hamilton–Norwood scale is utilized for MPHL, offering a detailed classification into various stages. For women, the Sinclair Scale or the Savin classification is applied, both of which assess the extent of hair thinning based on scalp density (York et al., 2020a). These methods allow for a precise evaluation without reliance on advanced imaging tools. When the diagnosis of AGA is not straightforward, a more in-depth analysis may be warranted, including the quantification of miniaturized hair, hair shaft diameter, and the density of hair follicles per unit of skin. In cases of diagnostic uncertainty, a scalp biopsy offers a conclusive diagnostic measure (Rakowska et al., 2009).

The genesis of AGA is rooted in a multifaceted array of influences, including genetic, epigenetic, hormonal, and environmental elements, with genetics being particularly influential. Comprehensive genomic scans, known as Genome-Wide Association Studies (GWAS), have uncovered a significant number of genetic markers—exceeding 190—that are associated with AGA, with the androgen receptor (AR) gene variants predominantly contributing to over 60% of the genetic predisposition (Heath et al., 2003). The conversion of testosterone (T) to Dihydrotestosterone (DHT) by 5α-reductase is a pivotal biochemical process in AGA, where DHT is identified as the principal mediator. DHT and T engage with the AR to initiate intracellular signaling cascades that modulate gene expression within the nucleus (Jain and De-Eknamkul, 2014). DHT’s binding affinity to AR and its signaling efficacy are substantially higher than that of T, highlighting its dominant role in the regulation of hair follicle biology (Inui and Itami, 2011).

In the hair growth cycle, the dermal papilla (DP) is a central regulator of hair follicle (HF) regeneration. Examinations of scalp tissue samples from individuals with AGA have shown a correlation between increased DHT levels and heightened AR expression in dermal papillary cells (DPCs), particularly in areas affected by hair loss. This contrasts with the normal expression levels in unaffected regions. Current research indicates that DHT can trigger DPCs to produce paracrine factors that downregulate the expression of growth-promoting factors such as insulin-like growth factor-1 (IGF-1), basic fibroblast growth factor (BFGF), and vascular endothelial growth factor (VEGF), while upregulating the expression of pro-apoptotic factors like transforming growth factor-β1 (TGF-β1), interleukin-1α (IL-1α), and tumor necrosis factor-α (TNF-α) (Rossi et al., 2016). This shift in the balance of these factors disrupts the growth phase of HFs, propelling them into a resting state and eventually leading to hair loss.

The Wnt/β-catenin signaling pathway is instrumental in the embryonic development and regeneration of hair follicles, promoting the onset of the growth phase. In contrast, the bone morphogenetic protein (BMP) signaling pathway exerts an inhibitory effect on the initiation of hair follicle growth. The delicate balance between these pathways is essential for the transition of HFs from the resting phase to the growth phase. In AGA, DHT has been shown to impede the proliferation of keratinocytes mediated by WNT3a and to suppress the Wnt/β-catenin signaling pathway (Heath et al., 2003). Furthermore, the activation of DHT and AR signaling can inhibit the canonical Wnt pathway (Premanand and Reena Rajkumari, 2018). This leads to the activation of glycogen synthase kinase-3β in DPCs, resulting in the degradation of β-catenin and, consequently, the inhibition of hair follicle stem cell (HFSC) differentiation in AGA. Additional studies have identified that DHT can downregulate Wnt agonists such as Wnt5a and Wnt10b, as well as the Wnt antagonist DKK-1 in DPCs (Leirós et al., 2017), ffurther disrupting the Wnt signaling pathway and affecting hair differentiation. The role of prostaglandins (PG) in hair growth is also significant. Elevated levels of prostaglandin D2 synthetase (PTGDS) and its metabolite PGD2 have been observed in the scalp of individuals with AGA, while the levels of PGE2 are found to be lower. Clinical evidence suggests that PGD2 has an inhibitory effect on hair growth, while PGE2 and F2α promote it. The miniaturization of hair follicles in AGA may be associated with increased PGD2 secretion and its interaction with the G protein-coupled receptor 44 in hair follicles, which may hinder the differentiation of HFSCs (Nieves and Garza, 2014; Garza et al., 2012).

### 2.2 Alopecia areata

AA is an autoimmune disease and the second most common type of alopecia after AGA; it affects more than 2% of the world’s population (Lee HH. et al., 2020). AA is characterized by sudden, non-scarring hair loss, with a higher incidence rate in individuals aged 10–25 years (about 60%); its incidence decreases with age. The etiology of AA includes genetic factors, environmental factors, oxidative stress, and other factors, among which genetic factors play a dominant role. Genetic epidemiological studies have shown an increased risk in first-degree relatives of patients with AA. The estimated lifetime risk for their siblings, parents, and offspring is 7.1%, 7.8%, and 5.7%, respectively, compared to 2% in the general population (Blaumeiser et al., 2006).

The clinical manifestations of AA vary greatly. The Severity of Alopecia Tool (SALT) is often used to evaluate the severity of AA. According to this method, the scalp area is divided into four parts, with each part representing the percentage (%) of the total scalp area: left side (18%), right side (18%), top (40%), and back (24%). The hair loss in each part is observed, judged, and scored, and then the total value is summed up, reaching a maximum of 100%. Based on the estimated SALT score, patients can be categorized into five subgroups: S0 (no hair loss), S1 (≤25% hair loss), S2 (25%–49% hair loss), S3 (50%–74% hair loss), S4 (75%–99% hair loss), and S5 (100% hair loss) (Olsen et al., 2004).

The Alopecia Areata Progress Index, developed in 2016, further divided AA based on the hair-pulling test and dermatoscopy results (Jang et al., 2016). A notable characteristic of AA is the presence of exclamation-mark-like hair, indicative of malnourishment. AA not only affects the scalp but also the eyebrows, body hair, and nails. Depending on the extent of AA, it can be broadly divided into three types: patchy alopecia (AF), which typically involves small, round, or patchy lesions on the scalp; alopecia totalis (AT), where hair loss occurs throughout the scalp; and alopecia universalis (AU), which involves hair loss on both the scalp and the body (Pratt et al., 2017). The disease courses of these three types of AA vary considerably. Patients with AF, the mildest form, can usually recover spontaneously within a year. However, more than 40% of patients with AF may have a relapse in the next few months, and more than 25% of patients may progress to more severe hair loss, potentially leading to AT. Even after complete hair regrowth, there is a 33% chance of recurrence within 6 months (Uchiyama et al., 2012). The self-healing rate for patients with AT and AU is less than 10%.

The underlying pathological mechanism of AA remains unclear. Patients with AA show infiltration of T lymphocyte subsets (CD56^+^NKG2D^+^NK cells) around the HFs in areas showing hair loss. HFs are immune-privileged sites and can prevent autoimmune responses against hypothetical autoantigens expressed within the follicles. Under normal physiological conditions, an immunosuppressive environment is formed in and around HFs, which can effectively inhibit the infiltration of T lymphocyte subsets at HF by downregulating the expression of histocompatibility complex (MHC) class I molecules in the HFs, inhibiting the surface molecules required to present self-antigens to NK cells, including CD8^+^ T lymphocytes. However, abnormal expression of MHC class I and class II molecules occurs in the pre-cortical area infiltrated by focal inflammatory cells (Pratt et al., 2017). Hence, it is generally believed that AA results from a breakdown of the immune privilege in HFs, caused by immune mechanisms.

Modern research indicates that IFN-γ secretion, the upregulation of NKG2D ligands (such as MICA and ULBP3/6), and the increased expression of MHC I and MHC II molecules and chemokines (such as IL-15, IL-2, and CXCLs) in HFs, all contribute to the failure of HF immune privilege (Paus and Bertolini, 2013). Further research proves that the intravenous injection of IFN-γ can induce AA-like hair loss in young C3H/HeJ mice, whereas mice deficient in IFN-γ do not show symptoms of AA-like hair loss, proving that IFN-γ plays an important role in the pathogenesis of AA. Moreover, CD8^+^NKG2D^+^T cells play an important role in the development of AA. They produce IFN-γ through the JAK1 and JAK2 pathways and stimulate follicular epithelial cells to generate IL-15, which can bind to the surface of CD8^+^NKG2D^+^T cells to further stimulate the production of IFN-γ through the JAK1 and JAK3 pathways, thereby forming a positive feedback loop. The use of JAK inhibitors (JAKi) can block the signal transduction of IFN-γ and effectively improve AA-like hair loss (Zhou et al., 2021). GWAS studies conducted on patients with AA have identified 14 genetic loci related to the condition, including *NKG2D* (an NK cell receptor), *NKG2DL3* (an NK cell ligand), and the Retinoic Acid Early Transcript 1L protein (encoded by the RAET1L gene, also known as ULBP6) (Carroll et al., 2002). These specific genetic factors are related to AA rather than other autoimmune diseases and play a key role in the pathogenesis of AA.

### 2.3 Trichotillomania

Trichotillomania is a condition characterized by the inability of patients to keep themselves from repeatedly pulling out their own hair, thus resulting in hair loss and functional impairment. It has an incidence of about 0.5%–2.0% (Grant and Chamberlain, 2016). It is one of the most common causes of temporary hair loss in children and is generally diagnosed as a mental illness in adults. Among adults suffering from trichotillomania, the ratio of female to male patients is 4:1, whereas the gender distribution among affected children are more evenly balanced (Lewin et al., 2008). The age of onset of typical trichotillomania is 10–13 years. Patients with trichotillomania usually unconsciously pull hair from any part of the body, most frequently from the scalp, eyebrows, and pubic area (Woods et al., 2006). This disorder is usually related to psychosocial dysfunction, low self-esteem, and social anxiety, and often occurs together with other disorders, such as major depression, anxiety, and substance use disorder. The clinical manifestations are alopecia patches with different sizes and irregular shapes. The hair around the alopecia area is often damaged, probably accompanied by hardened skin and small wounds caused by scratching. In chronic and severe cases, trichotillomania may lead to cicatricial alopecia (Alessandrini et al., 2021). The diagnosis of trichotillomania is often complicated by patients’ reluctance to disclose their hair-pulling habits due to factors such as embarrassment and shame. Therefore, it is frequently misdiagnosed as AA, obsessive-compulsive disorder, or other diseases. Trichotillomania is a chronic condition that may lead to serious consequences. For example, some patients eat their hair after pulling it out (“trichophagia”), which may lead to gastrointestinal obstructions known as “trichobezoars” and require surgery in extreme cases (Grant and Odlaug, 2008). Therefore, scalp biopsy is recommended in cases where trichotillomania is suspected.

Genetic factors may play a role in trichotillomania. Some family research reports indicate an increased incidence of trichotillomania in the first-degree relatives of those affected by the condition. Modern research (White et al., 2013) suggests that individuals with trichotillomania may suffer from neural circuit disorders related to inhibition of habitual actions and emotional regulation. Compared to individuals without the condition, those with trichotillomania show reduced volume of the left inferior frontal gyrus and an increased volume of the right wedge. In addition, the gray matter density of those with trichotillomania is higher in several brain regions (left caudate nucleus/putamen, left amygdala-hippocampus structure, left and right cingulate cortex and right frontal cortex) associated with emotional regulation, habit formation, and top-down cognition. Adults with trichotillomania have weakened nucleus accumbens response to reward expectations. However, these individuals show increased sensitivity to the outcomes of gains and losses.

### 2.4 Loose anagen hair syndrome

Loose anagen hair syndrome (LAHS) is characterized by a defect in the anchoring of hair to HFs. This condition can occur sporadically or be inherited in an autosomal dominant pattern hair disease and predominantly affects girls aged 2–6 years (Contin and Rocha, 2021). The clinical manifestations include diffused thinning of hair and irregular bald patches. In a tensile test, growing hair falls off easily, revealing a folded proximal stratum corneum, the absence of the inner root sheath, and a bent matrix (Alessandrini et al., 2021). Staining for citrulline, which is abundant in the inner root sheath, reveals that almost all of the shed hair is in the growth phase. While most cases of LAHS occur independently, it can also occur in individuals with genetic or developmental disorders, including vaginitis, Noonan syndrome, and hypohidrotic ectodermal dysplasia.

The key to diagnosing LAHS lies in a detailed examination of infants or young children with diffuse, non-scarring alopecia. Histological analysis from a scalp biopsy reveals that in LAHS, the outer root sheath is separated from the vitreous layer, and the inner root sheath is separated from the hair cuticle. The microscopic appearance of the hair is an important aspect of diagnostic evidence. One of the diagnostic criteria is that about 70% of the hair should be in a loose anagen (growth) phase. Recent research indicates that the external application of minoxidil shows promising improvement in this syndrome (Jerjen et al., 2021). In an analysis of the research conducted by Chapalain et al. (2002), it was observed that mutations in the gene encoding for the companion layer keratin (*K6HF*) were identified in some members of three out of nine families affected by Alopecia Simplex Syndrome (LAS). These findings suggest that the genetic underpinnings of LAS may extend beyond a single gene encoding for keratins expressed in the inner root sheath (IRS) or the companion layer, which is the innermost layer of the outer root sheath (ORS). Another potential candidate gene could be that encoding for a novel keratin (*K6IRS*) specific to the IRS (Porter et al., 2001). The defective keratinization process leads to a compromised adhesion between the ORS and IRS, which in turn results in a premature termination of the anagen phase, potentially accounting for the reduced hair length.

### 2.5 Anagen effluvium

Anagen effluvium refers to the condition where a large amount of hair in the growing phase falls out in a short time due to adverse effects of certain drugs, physical factors, or mental stress (Starace et al., 2022). It usually indicates hair loss caused by damage to HF mitosis or normal metabolism, such as hair loss caused by chemotherapy for head and neck cancer, the use of toxic drugs containing mercury, boron, thallium, etc. (York et al., 2020b) Conditions such as AA, pemphigus vulgaris, and severe HF malnutrition can also lead to anagen effluvium (Delmonte et al., 2000). Hair in the growing period is characterized by epithelial cell proliferation, in which bulb stromal cells show the greatest proliferation activity during hair shaft formation. When the mitotic activity is stopped or weakened due to sudden events, the keratinization of the proximal part of the hair shaft becomes impaired, and the hair tube becomes narrow and broken, thereby resulting in hair growth failure (Trüeb, 2007). Under normal conditions, about 90% of the scalp hair is in the growing stage. During anagen effluvium, only the proliferating cells in the bulb are affected, and the static stem cells in the bulb responsible for restarting HF growth are retained, making anagen effluvium usually reversible (Kanwar and Narang, 2013). The alopecia caused by chemotherapy presents as diffuse or patchy hair loss from the crown and sides of the head, depending on the amount of hair in the active growth stage. Anagen effluvium mostly occurs in the second week after starting treatment, and most patients experience hair regrowth within 1–3 months of discontinuation of chemotherapy (Contin et al., 2021). However, some radiation treatments may lead to permanent hair loss, possibly due to the inflammatory changes caused by continuous radiation, which gradually damages the HFSCs and may result in cicatricial alopecia. The hair-pulling test and microscopic hair observation are quite effective in diagnosing anagen effluvium. A conical fracture of the hair shaft is a characteristic feature of this condition. Pathological chemical tissue sections can be used to distinguish anagen effluvium from other types of hair loss. In a 4-mm scalp biopsy sample, having less than 15% of HFs in the cessation phase is a criterion to effectively distinguish anagen effluvium from TE. During anagen effluvium, there are no signs of inflammation, inner sheath dystrophy, or traction in the HFs, which can assist in distinguishing it from AA, AGA, and traction alopecia. At present, there is no specific treatment for anagen effluvium. The focus should be on identifying and removing the cause of hair loss, maintaining adequate nutrition, and managing stress to effectively alleviate the condition.

### 2.6 Telogen effluvium

TE is a common type of diffuse, scarless alopecia, which is more common in women. It is characterized by diffuse thinning of the scalp and lack of tiny hair follicles, particularly in middle-aged women (Stefanato, 2010). TE is usually divided into acute and short-term forms according to the duration of the condition. Acute TE often occurs after some acute injuries to the body, with potential causes including systemic diseases, drug use, fever, stress, weight loss, childbirth, iron deficiency, and inflammatory scalp diseases (Alessandrini et al., 2021). It is estimated that TE affects one-third to half of women after delivery and that elderly women are more likely to suffer from acute TE after experiencing high fever, surgery, or severe psychological stress (Chien Yin et al., 2021). Generally, acute TE gradually subsides within 6 months. However, some patients continue to experience hair loss beyond this period, with intermittent or spontaneous periods of remission and recurrence. This kind of alopecia is called chronic TE, which is more likely to occur in middle-aged women, although the exact causes remain unclear (Rebora, 2017). Acute TE can be diagnosed by assessing for sparseness in the temporal part and the edge of the frontal and occipital lobes, a combination called the “epidermal triad (Contin and Rocha, 2021).” For the diagnosis of chronic TE, a complete medical history and comprehensive clinical examination are usually required to exclude other causes. Routine examinations include a complete blood count and thyroid function tests. Syphilis serology, antinuclear antibody titer, serum zinc level, and other examinations are conducted if necessary (Chien Yin et al., 2021).

Headington’s classification of TE into five types has been considered less practical for clinical application (Grover and Khurana, 2013). HFs undergo a cyclical phase of active growth, degeneration, and rest. Hair loss occurring at the end of the resting phase is termed teloptosis, which is independent from the beginning of the next growth phase. Teloptosis is usually caused by the loss of adhesion between the stick hair cells and their epithelial envelope cells. It is usually possible to judge whether the hair is in the early cessation period or teloptosis according to the presence or absence of the upper sheath (Chien Yin et al., 2021). Currently, the simpler Rebora method is usually used to classify TE into three categories: premature teloptosis, collective teloptosis, and premature entry into the telogen phase. Premature teloptosis alopecia is generally caused by local inflammation. External application of drugs containing all-trans retinoic acid and salicylic acid, external application of minoxidil, or excessive ultraviolet radiation may also lead to premature teloptosis, which may be caused by the decomposition of cadherin through proteolysis and the consequent release of exogenous hair. Collective teloptosis is more common in newborns, *postpartum* women, and those with long-term use of estrogen-containing drugs. Premature entry into the telogen phase may be a response to cell-inhibitory drugs, nutritional deficiencies, or lymphocytotoxic (autoimmune) activities (Chien Yin et al., 2021).

## 3 Treatment of common non-cicatricial alopecia

### 3.1 Common western medicine

#### 3.1.1 Minoxidil

After its topical application, minoxidil is converted into minoxidil sulfate by S-transferase in the outer root sheath of HFs. Minoxidil sulfate acts as an adenosine triphosphate-sensitive potassium channel opener, which effectively dilates arteries by relaxing vascular smooth muscle and relaxes the muscle tissue on the capillary walls of the scalp. This relaxation facilitates increased blood flow to the scalp and HFs, promoting hair growth. Therefore, drugs such as retinoic acid, which increase the expression of thiotransferase, can enhance the efficacy of minoxidil (Chien Yin et al., 2021).

The FDA has approved the use of 2% and 5% minoxidil to treat male AGA, mainly for external application. In addition, 2% minoxidil is also the only drug approved by the FDA to treat female hair loss. Research on the mechanism of minoxidil for promoting hair growth shows that it can shorten the telogen phase by prolonging the anagen (growing) phase of HFs, promote the mitosis of epithelial keratinocytes, and activate the Wnt/β-catenin signal pathway in DPCs to promote hair growth. Minoxidil is more effective for patients with a considerable amount of hair, a short duration of hair loss, and a small bald area. Its efficacy is poor for patients with severe AGA. In clinical trials, it has been found that 5% minoxidil solution is more effective than 2% minoxidil solution in alleviating hair loss. However, the effectiveness of a higher dose of minoxidil (10%) for external use is lower than that of a 5% minoxidil solution (Ghonemy et al., 2021). In recent years, oral and sublingual administrations of minoxidil have also received increased attention compared to traditional topical application. Numerous clinical trials have shown that the efficacy of oral administration of 0.25 g/d of minoxidil is comparable to the topical application of 2% minoxidil, and the efficacy of oral administration of 1 g/d of minoxidil is comparable to the topical application of 5% minoxidil. However, oral administration tends to cause more significant side effects compared to topical application. Although the side effects are described as mild without any significant impact, further large-scale long-term clinical trials are needed to verify its safety. Compared to oral administration, which has low bioavailability due to the first-pass effect of the liver, sublingual administration of minoxidil, which does not involve the first-pass effect, offers higher bioavailability and lower side effects, suggesting that sublingual administration may be a potentially more effective method of administration. A phase 1B clinical trial discovered that during the 24-week treatment period, sublingual administration of 0.45–1.35 mg/d of minoxidil could promote hair growth in a dose-dependent manner, indicating that sublingual administration of minoxidil might be a relatively superior method of administration. However, further clinical trials are necessary to determine the optimal dosage of minoxidil for sublingual administration and to evaluate its safety.

Although minoxidil is effective in alleviating alopecia, it is usually accompanied by adverse reactions such as generalized hirsutism, itchy skin, and rapid heartbeat, as well as drug dependence, where alopecia reappears after drug withdrawal. Therefore, modern research often combines minoxidil with other therapeutic drugs or methods to reduce side effects and improve its effectiveness. The related research studies conducted in this year are summarized in Supplementary Table S1.

#### 3.1.2 Finasteride

Finasteride 1 mg daily is FDA-approved for the treatment of MPHL. Finasteride competitively inhibits the human type II 5α-reductase (5AR) enzyme, which is crucial in the conversion of T to DHT. There are two isoenzymes of 5α-reductase: type I and type II. Type I is mainly distributed in the liver, kidney, sebaceous glands, and brain and has reproductive activity. Type II mainly exists in the gonads and HFs of the scalp. Finasteride combines with type II 5α-reductase to form a stable enzyme complex to prevent the conversion of T to DHT, leading to the reduction of DHT levels in the scalp and serum, thereby effectively preventing androgen-dependent HF miniaturization (York et al., 2020b). Clinical studies have shown that finasteride needs to be applied continuously to maintain its curative effect. However, it has been associated with significant side effects on male sperm and libido (Blumeyer et al., 2011). Therefore, its use in females, especially pregnant women, should be exercised with caution (Rossi et al., 2012).

#### 3.1.3 Baricitinib

In AA, IFN-γ is generally considered the main immune factor leading to hair loss. It mainly acts on the IFN-γ receptors of HF epithelial cells, and these receptors are dependent on the activation of the JAK/STAT signaling pathway (Strazzulla et al., 2018). Therefore, modern research believes that blocking the signal transduction of the JAK-STAT signal pathway could interrupt the cytokine signaling related to the pathogenesis of AA, thus alleviating its symptoms. Baricitinib is a reversible competitive small molecule inhibitor in the JAK family (Xing et al., 2014), which selectively inhibits the expression of JAK1, JAK2, and TYK2. Its selectivity for JAK1 and JAK2 is about 100 times greater than that for JAK3. This lower affinity for JAK3 may potentially reduce the expected immunosuppressive effects of JAK3 inhibition. On 13 June 2022, the FDA approved JAK inhibitor baricitinib for the treatment of adult AA (Mogul et al., 2019), which was the first time that the FDA had approved systemic therapy for AA rather than treatments targeting specific parts of the body.

Baricitinib is now available in China (Wu et al., 2023), Japan, and many other countries for the treatment of AA, rheumatoid arthritis, specific forms of dermatitis, and other inflammatory conditions. Its mechanism of action involves the inhibition of intracellular signaling pathways. When the IFN-γ receptor receives a signal, baricitinib prevents the phosphorylation of the JAK enzyme located in the cell, leading to the failure of intracellular signal transduction, effectively blocking the IFN-γ signal pathway that regulates immune response, thereby inhibiting inflammatory responses. Baricitinib is mainly administrated orally and has an oral bioavailability of about 80%, with a half-life of about 12 h (Fasinu et al., 2011). Even though only a minor portion (6%) of baricitinib undergoes metabolism via CYP3A4, concurrent use with ketoconazole or rifampin did not result in a significant effect on the pharmacokinetics of baricitinib (Jorgensen et al., 2020).

In a randomized controlled phase II clinical trial on the efficacy and safety of baricitinib in treating adult AA, it was found that in the 12th week, patients with severe AA showed better outcomes when treated with 2 mg or 4 mg of baricitinib per day compared to those receiving 1 mg of baricitinib or a placebo (Jabbari et al., 2015). By the 36th week of treatment, the efficacy of both doses of baricitinib was better than that of the placebo. In addition, baricitinib was found to promote the growth of eyebrows and eyelashes (King et al., 2021). Further, several phase III clinical trials involving patients with severe AA revealed that hair regeneration in the 36th and 52 nd weeks after oral administration of baricitinib was better than that that after oral administration of a placebo, and the curative effect of 4 mg/d of baricitinib was more significant compared to the 2 mg/d dosage (King et al., 2022). However, these trials also reported some adverse reactions, the most common being upper respiratory tract infections, headaches, nasopharyngitis, acne, urinary tract infections, and elevated levels of creatine phosphokinase. These results suggest that baricitinib is an effective drug for the treatment of moderate to severe AA. However, longer-term clinical trials are needed to prove its long-term efficacy and safety.

#### 3.1.4 Ritlecitinib

Ritlecitinib is a selective JAK3 inhibitor and the inhibitor of the TEC family protein tyrosine kinases, which are expressed in hepatocellular carcinoma (Eisman and Sinclair, 2021). It has been approved by the US FDA for the treatment of AA, particularly for moderate to severe cases (Gupta et al., 2023). Ritlecitinib can irreversibly and covalently bind to Cys-909 in JAK3. *In vitro* experiments have indicated that its half-maximal inhibitory concentration (IC_50_) on JAK3 is 33.1 nM, whereas its inhibitory effect on JAK1, JAK2, and TYK2 is more than 10,000 nM, demonstrating its high specificity for JAK3. Therefore, its mechanism of action displays that it only inhibits γc-chain receptor signals (IL-15 and IL-21) and avoids affecting the signal transduction of JAK1-dependent cytokines such as IL-2, IL-6, IL-7, IL-9, etc., thus reducing the occurrence of adverse reactions such as respiratory tract infections and infectious diarrhea (Ramírez-Marín and Tosti, 2022). Ritlecitinib can inhibit the killing function of CD8^+^T cells and NK cells by inhibiting the TEC enzyme. *In vitro* experiments have demonstrated the ability of ritlecitinib to inhibit the differentiation and function of Th1 and Th17 cells. Ritlecitinib can inhibit the phosphorylation of STAT5 induced by enzymes such as IL-2, IL-4, IL-7, and IL-15, as well as the phosphorylation of STAT3 induced by IL-21. These processes are all closely related to AA, and the results indirectly verify that ritlecitinib can be used to treat AA.

Owing to its effective gastric absorption, ritlecitinib is mainly administrated orally. The recommended daily dose in clinical trials is 50 mg, and the maximum dose is 200 mg per day. In a randomized, double-blind, placebo-controlled phase 2a multicenter clinical trial, changes in molecular biomarkers were observed in patients with AA with moderate to severe scalp alopecia (>50% of the scalp) treated with ritlecitinib. After 12 weeks of administration, a significant improvement was observed, with the 24th week showing even stronger effects. The efficacy was more than 100% in the transcriptional group of scalp lesions, developing toward the direction of non-lesion scalp areas. Greater improvement was found in patients after 12-week baricitinib treatment, whereas a substantially greater overall improvement was observed after 24-week ritlecitinib treatment (Guttman-Yassky et al., 2022). Other similar clinical trials also confirmed that baricitinib has better treatment performance compared to placebo (Guttman-Yassky et al., 2022; Winnette et al., 2022). According to two clinical safety trials with sample sizes of 715 and 142 patients with AA, common adverse events reported by patients receiving a 200-mg loading dose followed by 50 mg of ritlecitinib daily include nasopharyngitis (14.0% [25/179]), upper respiratory tract infection (12.3% [22/179]), headache (12.8% [23/179]), acne (6.1% [11/179]), diarrhea (7.3% [13/179]), and nausea (7.8% [14/179]). In the first study, no serious adverse events occurred in more than one patient, and no dose-dependent adverse reactions were reported. The proportions of patients reporting non-severe and severe adverse events in all groups ranged from 51.6% to 64.1% and 0.8%–4.6%, respectively. Considering the short duration of safety assessments, longer-term clinical trials are recommended for a more comprehensive evaluation.

#### 3.1.5 Other therapeutic drugs

Dutasteride is a 5α-reductase inhibitor with relative specificity, whose inhibitory effects on type I and II enzymes are 3 times and 100 times greater, respectively, than those of finasteride (Stough, 2007). Dutasteride at a dose of 0.5 mg/d can effectively reduce the levels of DHT in serum (Clark et al., 2004). However, compared with finasteride, its half-life in serum is longer. Consequently, effects such as reduced sperm count and libido may persist for a long time after discontinuing the drug. If taken by pregnant women, it may lead to fetal malformation; therefore, the FDA has not approved dutasteride for hair loss treatment at present (Ghonemy et al., 2021).

Bimatoprost is a synthetic prostaglandin E2 (PGE2) analog. It is known that PGs play an important role in the regulation of HC. Administration of bimatoprost can extend the hair growth phase, leading to increased hair length. In addition, it can accelerate the growth of new hair and slow down the shedding of existing hair (Barrón-Hernández and Tosti, 2017). Recent studies have shown that externally applying a 0.03% bimatoprost lotion daily for 12–16 weeks can significantly increase the diameter and number of HFs.

### 3.2 Treatment using Chinese medicine

**FIGURE 2:**
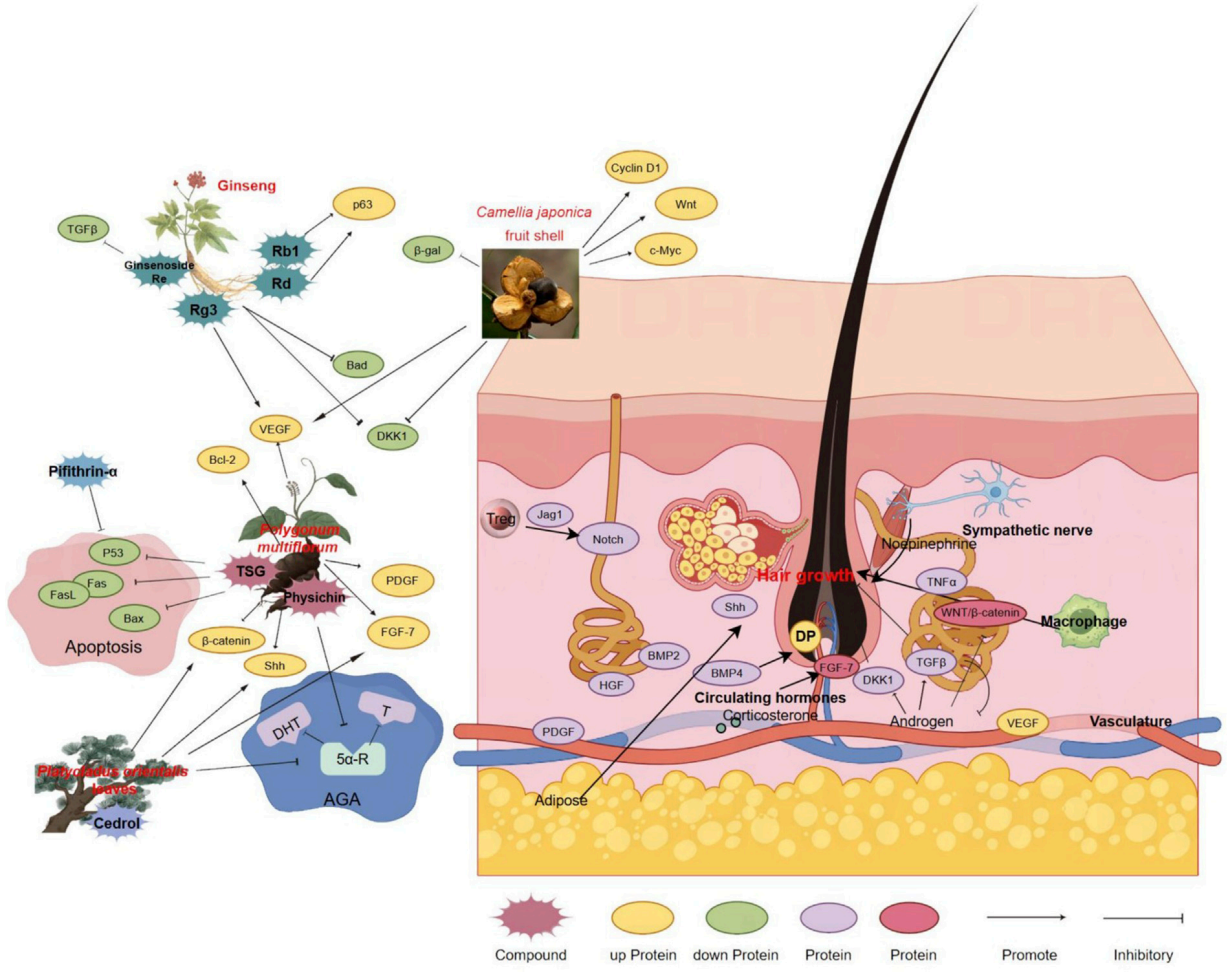
Therapeutic Mechanisms of Chinese Medicine in Hair Restoration. This figure provides a comprehensive illustration of the role of traditional Chinese medicine (TCM) in treating various forms of hair loss, each characterized by distinct pathophysiological processes. It showcases the intricate network of Chinese medicinal herbs and their active components, along with their proposed effects on promoting hair growth and inhibiting apoptosis. Key signaling pathways involved in the hair growth cycle, particularly those regulating the balance between anagen and telogen phases, are highlighted. The figure also depicts how Chinese medicine influences proteins related to hair follicle cycling and the overall hair growth process. Arrows indicate the direction of effects and the interplay between different components of the treatment. The figure was created using Figdraw (www.figdraw.com).

The pursuit of efficacious treatments for hair loss is increasingly embracing traditional Chinese medicine, recognized for its holistic approach and historical efficacy. Figure 2 delineates the therapeutic mechanisms of Chinese medicine in hair restoration, illustrating its application across diverse hair loss etiologies. The figure emphasizes the modulation of signaling pathways integral to the hair growth cycle, specifically the transition between anagen and telogen phases, and the influence on proteins critical to follicle cycling. The intricate interplay of components within the Chinese medicine framework underscores its substantial impact on advancing hair restoration research.

#### 3.2.1 *Polygonum multiflorum* Thunb

*Polygonum multiflorum* Thunb., the tuberous root of *Pleuropterus multiflorus* (Thunb.) Nakai, is widely used in China and East Asia for its efficacy in improving blood essence and for darkening beard and hair. It is widely applied in traditional practices to treat hair loss and graying hair. Recent research has revealed that the topical application of *P. multiflorum* Thunb. extract to the epidermis of C57BL6/N mice can upregulate the expression of β-catenin and Sonic Hedgehog (Shh), which are mostly expressed in the anagen phase. In addition, more and larger HFs were observed, which indicates that the topical application of *P. multiflorum* Thunb. extract can promote hair growth by inducing the anagen phase (Sun et al., 2013; Cui et al., 2011). Oral administration of *P. multiflorum* Thunb. extract has also been found to promote hair growth, potentially mediated by the promotion of fibroblast growth factor-7 (Li et al., 2015). *In vitro* experiments have confirmed that the *P. multiflorum* Thunb. extract can stimulate the proliferation of human DPCs, enhance mitochondrial activity, and increase the expression of anti-apoptotic protein Bcl-2 in DPC cultures and the expression of PDGF and VEGF, while inhibiting the expression of the pro-apoptotic protein Bad and the telogen stage-induced protein Dkk-1. The extract also shows obvious antiandrogenic effects, such as inhibiting AR expression and reversing DPC spheroid shrinkage caused by DHT treatment, indicating that *P. multiflorum* Thunb. could be used as a natural remedy to treat AGA (Shin et al., 2020). The main active ingredients in *P. multiflorum* Thunb., namely, emodin (physichin) and trans stilbene glycoside (TSG), also promote hair growth. The structure of physichin is similar to that of 5α-reductase inhibitors and can significantly reduce the concentration of DHT in the skin without changing serum DHT levels or T levels in the skin and serum, suggesting its potential as a natural active ingredient for the treatment of AGA (Lao et al., 2022). TSG can inhibit Fas-induced exogenous apoptosis, Bax-induced endogenous apoptosis, and p53 expression, consequently delaying the start of the HF telogen phase, thereby promoting hair recovery and growth (Chen et al., 2018).

Studies have confirmed that the compound Shi-Bi-Man, mainly composed of *P. multiflorum* Thunb., ginseng, angelica sinensis, and other medicinal ingredients, can activate resting HFs and promote new hair growth. TSG and (−)-epigallocatechin gallate are the main active components, indicating that TSG can be used as a natural active ingredient for hair loss treatment (Han et al., 2022). Other ingredients in *P. multiflorum* Thunb., such as torachrysone-8-O-b-D-glucoside and (E)-2,3,5,4′-tetrahydroxysilbene-2-O-β-D-glucoside, have also been shown to promote the proliferation of DPCs (Sun et al., 2013).

In summary, *P. multiflorum* Thunb. may be an effective natural remedy for alopecia. Due to its multi-component and multi-target mechanism of action, it could be useful in treating a range of hair loss conditions, including AGA and AA. However, further research is necessary to fully understand its efficacy and scope of application.

#### 3.2.2 Ginseng

Ginseng, a perennial herb from the Panax genus in the Araliaceae family, is widely used in traditional medicine for its ability to treat various diseases by reinforcing vital Qi, tonifying qi, controlling blood, restoring pulse, and preventing prostration. Modern research shows that ginseng is also effective for alopecia (Choi, 2018). Ginseng extract has been shown to improve the survival rate of DPCs by increasing the ratio of the anti-apoptotic molecule Bcl-2 to pro-apoptotic molecule Bax in DPCs, showing effects similar to those shown by minoxidil. Notably, 1 mg/mL of ginseng extract exhibits a stronger effect compared to 5% minoxidil in promoting the entry and extension of the hair growth phase (Park et al., 2011). *In vivo* and *in vitro* experiments indicate that ginseng extract can inhibit the effects of Dkk-1, indicating its anti-apoptotic capabilities and potential to promote hair growth (Lee et al., 2017). Clinically, herbal extracts containing ginseng have shown similar therapeutic effects to those of 3% minoxidil, positioning it as an alternative therapy for AGA (Lueangarun and Panchaprateep, 2020). Korean ginseng has also been confirmed to improve hair regeneration in patients with AA (Oh and Son, 2012). Red ginseng oil, extracted from red ginseng, improves hair growth by reducing the protein level of TGF-β and enhancing the expression of anti-apoptotic protein Bcl-2. It is effective in treating AGA and protecting the skin from ultraviolet radiation (Truong et al., 2021). Ginseng contains many active components, among which ginsenosides play a critical role. Ginsenoside components can be divided into protopanaxadiol (PPD) and protopanaxatriol (PPT) groups. PPD components such as ginsenoside Rb_1_ and Rd can promote hair growth via p63-inducing HF keratinocytes. Ginsenoside Rg3 increases the expression of VEGF in rat HFs and DP, and enhances hair growth by stimulating HFSCs (Shin et al., 2014). Ginsenoside Re in PPT selectively inhibits TGF-β signaling to promote hair growth (Li et al., 2016). Other ingredients in ginseng, such as gintonin, promote mouse hair growth by inducing instantaneous [Ca^2+^]i and stimulating the proliferation of DPCs in HFs (Lee NE. et al., 2020). The evidence suggests that ginseng extract and its various active ingredients are effective in improving alopecia and could be further developed as candidate drugs for treating various types of alopecia.

#### 3.2.3 *Platycladus orientalis(*L.)Franco leaves

*Platycladus orientalis* (L.)Franco leaves, the dried branches and leaves of the *P. orientalis* plant, are traditionally used as they cool blood, stop bleeding, promote hair growth, and darken hair. Folk remedies commonly use them to treat external bleeding, lung heat cough, white hair, and hair loss. Modern research has found that a decoction of *P. orientalis* leaves can induce the expression of β-catenin and SHH proteins in C57BL/6N mice, mediating the early entry of HFs into the anagen phase (Shan et al., 2014). Other studies have shown that the extract of *P.orientalis* leaves is an effective 5α-reductase inhibitor, which can inhibit the activity of 5α-reductase on the skin, thereby reducing DHT levels without affecting androgen levels in the blood. The extract can also promote the proliferation of DPCs and prolong the anagen phase of HFs, thereby promoting hair growth. The volatile oil component of the leaves plays a significant role in this process. *In vivo* experiments on C57BL/6 mice have shown that cedrol, an active ingredient in the volatile oil of *P. orientalis* leaves, exhibits a better effect in promoting hair growth compared to 2% minoxidil. It also demonstrates a highly selective effect on promoting hair growth in female mice (Zhang et al., 2019).

#### 3.2.4 *Camellia japonica* L. *(C.Japonica)* fruit shell

The fruit shell of *Camellia japonica* L. *(C.japonica)*, commonly discarded, has its biological activity yet to be fully explored. A recent investigation has delved into the potential of the *C. japonica* fruit shell extract (CJFSE) in ameliorating hair loss (You et al., 2023). The study demonstrated that CJFSE significantly enhanced the proliferation rate and the size of spheroids in human follicle dermal papilla cells (HFDPCs). Additionally, CJFSE induced an upregulation in the expression levels of key growth factors such as VEGF-A, Wnt-1, c-Myc, and Cyclin D1. Furthermore, the extract exhibited an inhibitory effect on 5α-reductase activity, counteracting the dihydrotestosterone (DHT)-induced reduction in cell proliferation, the secretion of Dickkopf-1 (Dkk-1), and the activity of β-galactosidase (β-gal). CJFSE also demonstrated a notable DPPH radical scavenging activity and mitigated the reactive oxygen species (ROS) production induced by hydrogen peroxide, as well as the associated β-gal activity. The chemical composition analysis of CJFSE via high-performance liquid chromatography (HPLC) revealed the presence of gallic acid and protocatechuic acid, which may contribute to the observed biological activities. These findings suggest that CJFSE could be a promising candidate for hair loss treatment, warranting further research into its mechanisms of action and potential applications in dermatological therapies.

The aggregate findings from these observations place CJFSE as a candidate of interest for further exploration of its role in alleviating hair loss. Subsequent studies should endeavor to delineate the specific molecular pathways modulated by CJFSE and to evaluate its efficacy and safety in clinical applications.

#### 3.2.5 Others

The research on the treatment of non-scarring alopecia using other herbal plants and their extracted components is shown in Supplementary Table S2.

## 4 Discussion

Alopecia, a complex dermatological condition, exerts a profound impact on affected individuals, extending beyond physical appearance to encompass social and psychological ramifications. The spectrum of alopecia’s effects includes the potential for diminished social status and the imposition of a considerable psychological burden, with patients often grappling with anxiety, depression, and in severe cases, mental illness. Clinically, addressing the psychological impact of alopecia is as crucial as managing the physical symptoms. Physicians should consider integrating psychological counseling and supportive therapy as part of a comprehensive treatment strategy.

In non-cicatricial alopecia, genetic predispositions are frequently at the forefront, yet the interplay of modern life’s accelerated pace and escalating social pressures has brought to light the increasing prevalence of stress-induced hair loss conditions such as alopecia areata, trichotillomania, and telogen effluvium. This trend is observed across younger demographics, suggesting a shift in clinical focus towards early prevention and intervention. Notably, research has implicated the necessity of glucocorticoid signaling in regulatory T cells for hair growth (Liu et al., 2022), thereby highlighting the pivotal role of glucocorticoids in the pathophysiology of alopecia.

The therapeutic landscape for alopecia is marked by a concerted effort to develop novel pharmaceuticals, exemplified by 5α-reductase inhibitors for androgenic alopecia and JAK pathway inhibitors for alopecia areata. While the recent approval of ritlecitinib and baricitinib has provided new therapeutic options, their long-term safety profiles remain uncertain, necessitating post-marketing surveillance and real-world data analysis. FDA-approved treatments, including minoxidil and finasteride, while efficacious, are not without their side effects. Patients often face challenges with adherence due to adverse effects such as scalp irritation, dizziness, and hormonal disturbances, highlighting the need for improved formulations with enhanced tolerability.

Adjunctive treatment modalities, including microneedle therapy, low-level laser therapy (LLLT), and platelet-rich plasma (PRP) therapy, are designed to augment drug absorption or enhance efficacy through the release of growth factors and the facilitation of synergistic drug combinations. Cutting-edge approaches, such as the transplantation of cultured hair follicles and skin *in vitro* or the transplantation of autologous fat-derived stromal vascular fraction (SVF), aim to minimize invasiveness. However, their high cost and the need for specialized techniques limit their widespread clinical application, making them less accessible to the general population.

Traditional Chinese medicine (TCM) offers a distinct perspective, positing that hair is nourished by blood and reflects kidney function. TCM approaches to hair loss typically involve treatments aimed at nourishing the liver and kidneys and promoting the circulation of qi and blood. Despite their potential benefits, the clinical acceptance of TCM remains limited due to variability in formulation, lack of standardized clinical trials, and slower onset of action compared to conventional pharmaceuticals. Future research should focus on pharmacokinetics, optimizing delivery methods, and conducting large-scale clinical trials to validate efficacy and safety.

The imperative for the development of novel and more effective treatment options is underscored by the escalating prevalence of hair loss. To bridge the gap between research and clinical practice, future studies should prioritize comparative effectiveness research, personalized medicine approaches, and patient-reported outcomes to guide evidence-based treatment strategies. Ensuring that therapeutic efficacy is not compromised while adhering to the rigorous standards of pharmacological research will be crucial for advancing alopecia management.

## 5 Conclusion

Alopecia is a multifactorial condition influenced by genetic, environmental, and psychological factors. This review emphasizes the rising prevalence of stress-related alopecia, such as alopecia areata and telogen effluvium, highlighting the need for treatments that address both biological and psychosocial aspects.

Currently, FDA-approved treatments like minoxidil and finasteride, along with novel therapies such as JAK inhibitors, show promise. However, their long-term safety and efficacy require further validation. Adjunctive therapies like microneedling and PRP therapy have potential but face challenges in cost, accessibility, and patient variability.

Traditional Chinese Medicine (TCM) offers a more affordable approach with fewer side effects, but its slower onset and lack of standardized evidence limit widespread use. Future research should focus on optimizing these therapies, including their integration with modern treatments, and conducting large-scale clinical trials.

Looking ahead, personalized treatment approaches, including multi-target therapies and regenerative medicine, hold promise for improving long-term outcomes. More real-world data and longitudinal studies are needed to assess the safety and efficacy of emerging treatments.

In conclusion, while progress has been made in understanding alopecia and its treatments, there remains a need for continued research to develop safer, more effective, and personalized therapeutic options.
