# Supplementary material for: Research progress in the treatment of non-scarring alopecia: mechanism and treatment
Source: Front Pharmacol. 2025 May 23;16:1544068. doi: 10.3389/fphar.2025.1544068 (PMC12141338; doi:10.3389/fphar.2025.1544068)
Supplement: Supplementary file 1 [file Supplementaryfile1.docx]

Supplementary Material

**Table 1 Research results on the treatment of Minoxidil-Related.**

| **Dosage/ Regimen** | **Disease** | **Number of participants** | **Results** | **Adverse Effects** | **Author** |
| --- | --- | --- | --- | --- | --- |
| PRP injections in combination with applications of 5% minoxidil solution | AGA  （presence of AGA degrees I-IV on the Norwood-Hamilton scale inclusively） | Total n=69  （69M，0F）  mean age 29.7 ± 1.9 years | complex therapy exceeds the effectiveness of traditional therapy with minoxidil or PRP in terms of hair density the thickness of the hair shafts，the share of telogen hair , the share of vellus hair.(4 months) | No reported adverse effects | (1) |
| topical minoxidil 5% gel and spironolactone gel 1% | AGA  The duration of the disease ranged from 3 months to 10 years | Total n=60  （39M，21F）  aged 18–45 years | combined topical spironolactone 1% and minoxidil 5% gel group, clinical improvement were in 100% of the patients, eight of them have excellent response, ten have good response, and two patients have fair response. (12-month) | The more frequent side effect in patients was contact dermatitis, mainly presented as pruritus, burning and scaling in 20% of the patients. Most of the symptoms were mild and endurable.( No further details) | (2) |
| topical minoxidil 2%/d + microneedling/week | female pattern hair loss | Total n=20  （0M，20F）  aged 18–50 years | Effective n=17  Ineffective n=3  Ineffective=85%  significantly higher than that of the control group (45%) | All of the adverse reactions observed during the treatment period were mild. No severe adverse event was observed in either group. ( No further details) | (3) |
| intradermal injections of 0.5% minoxidil  (1 times/week, 10 consecutive weeks) | Female androgenetic alopecia (FAGA) | Total n=54  （0M，54F）  aged 18–65 years | Intradermal injections with minoxidil were more effective than placebo (P < .001) in the treatment of female androgenetic alopecia with a good safety profile.  it increased from 69.9%±1.9% before treatmentto 78.5%±2.0% after treatment. | headache in 5.6% (3/54)  burning in 11.1% (6/54)  itching in 11.1% of patients (6/54). | (4) |
| 5% topical minoxidil and nano-microneedle-assisted fibroblast growth factor | Male androgenic alopecia (MAA) | Total n=40  （40M，0F）  aged 22–50 years | combination therapy is superior to monotherapies in terms of hair density, hair diameter, and follicular unit density. | Three participants developed mild erythema, which disappeared within 24 h. | (5) |
| 5% topical minoxidil(1ml 5%) and low-level laser therapy (LLLT)-12min left half was turned on (Active) and the right half off (Sham) (2 times/d,6 months) | AGA  the degree of alopecia ranged from III vertex to VI (median IV ± 2). | Total n=21  （21M，0F）  The mean age of participants was 41.67 ± 6.76 years (range: 28–53 years) | both the Active and Sham sides exhibited an increase in total hair count, as well as terminal and vellus hair counts at 3 and 6 months, with no statistically significant difference detected between sides ( *P* > 0.05). | No reported adverse effects | (6) |

**Table 2 Research results on the treatment of non-scarring alopecia with other herbal plants and their extracted components.**

| Dosage/ Regimen | Model | Type | Results | Adverse Effects | Author |
| --- | --- | --- | --- | --- | --- |
| E. alba 2.5%  A. radix 2.5%  P. ginseng 2.5%  （Methanol extract） | no | Athymic Balb/c male nude mice  （7 weeks of age） | E. alba extract can significantly promote hair growth;  Manifested as an increase in hair density and length, an increase in the number of hair follicles (HFs), and an increase in follicular keratinocytes | No reported adverse effects | (7) |
| Alcohol extract from Vernonia anthelmintica willd (L.) 80 mg/kg/d | chronic restraint stress-hair loss model | male C57BL/6 mice  (5~6 weeks old, weighing 18-22 g) | AVE counteract stress-induced hair follicle growth inhibition in C57BL/6 mice in vivo and in vitro;  AVE counteract murine hair follicle growth inhibition caused by chronic restraint stress via inducing the conversion of telogen to anagen and inhibiting catagen premature | No reported adverse effects | (8) |
| extract of Centipeda minima (L.) A. Braun & Asch | no | Human hair follicle dermal papilla cells | extract of Cetipeda minima (CMX) induced the proliferation of HFDPCs  Wnt family member 5a (Wnt5a), frizzled receptor (FZDR), and vascular endothelial growth factor (VEGF) was upregulated;  The phosphorylation of ERK and JNK was enhanced by CMX in HFDPCs, and β-catenin accumulated significantly in a dose-dependent manner. | No reported adverse effects | (9) |
| Leaves of guava (Psidium guajava L.) (70% ethyl alcohol extract) | no | hair follicle dermal papilla cells (HFDPC);  human prostate cancer cells (DU-145) | The extract could minimize hair loss by inhibiting the synthesis of a potent androgen (dihydrotestosterone) | No reported adverse effects | (10) |
| Glucosinlates-enriched Brassica oleracea L.var.italic Planch extract (BOE) | AGA model(  Testosterone alone ,50 μg/ml) | Male C57BL/6 mice (6–8 weeks; Dermal papilla (DP) cells and HaCaT cells | BOE can promote the growth of hair follicle of mice;  BOE can prevent the testosterone-induced inhibition of dermal papilla (DP) cells viability and HaCaT cells viability;  BOE up-regulate the cytokeratin gene expression in HaCaT cells, prevent the increase in Bax gene levels induced by testosterone in DP. | No reported adverse effects | (11) |
| G. florida fruit extract (GFFE) (extract) | no | Male five-week-old C57BL/6 mice  human dermal papilla cells, hDPCs | GFFE showed strong hair growth-promoting activity;  GFFE could induce VEGF and Wnt/β-catenin signaling pathway activity while attenuating TGF-β1 synthesis activity in hDPCs and also found that GFFE up-regulated the protein expressions of VEGF and β-catenin and downregulated TGF-β. | No reported adverse effects | (12) |
| Cacumen Platycladi (CP) (extract) | no | Female C57BL/6 mice 7 weeks of age  Dermal papilla cells (DPCs) | The study showed that the extracts of CP could promote the proliferation of DPCs, and the activity of volatile oil was the best. | No reported adverse effects | (13) |
| Ancocyanin-rich blackcurrant extract (BCE) | animal models of FPHL  Ovariectomized(OVX) | OVX female Sprague–Dawley and sham surgery rats (12 weeks of age) | BCE has phytoestrogen activity in hair follicles and contributes to the alleviation of hair loss in a menopausal model in rats. | No reported adverse effects | (14) |
| Morus alba. L root extract(MARE) 20 wt% | no | human dermal fibroblasts (HDFs)  human umbilical vein endothelial cells (HUVECs)  hair follicle dermal papilla cells (HFDPCs) | MARE can stimulate the secretion of pro-proliferative and pro-angiogenic paracrine factors from HDFs, MARE has therapeutic potential as a hair loss preventative. | No reported adverse effects | (15) |
| 2% Angelica gigas Nakai (AGN) root extract (98% ethanol extract) | no | Male C57/BL6 mice  age, 6–7 weeks; body weight, 25 ± 2 g | AGE root extract promotes hair growth by regulating pro- and/or anti-inflammatory cytokines. | No reported adverse effects | (16) |

**References**

1. Pakhomova EE, Smirnova IO. Comparative Evaluation of the Clinical Efficacy of PRP-Therapy, Minoxidil, and Their Combination with Immunohistochemical Study of the Dynamics of Cell Proliferation in the Treatment of Men with Androgenetic Alopecia. Int J Mol Sci. 2020 Sep 6;21(18):6516.

2. Abdel-Raouf H, Aly UF, Medhat W, Ahmed SS, Abdel-Aziz RTA. A novel topical combination of minoxidil and spironolactone for androgenetic alopecia: Clinical, histopathological, and physicochemical study. Dermatol Ther. 2021 Jan;34(1):e14678.

3. Zhang Y, Sheng Y, Zeng Y, Hu R, Zhao J, Wang W, et al. Randomized trial of microneedling combined with 2% minoxidil topical solution for the treatment of female pattern hair loss in a Chinese population. J Cosmet Dermatol. 2022 Dec;21(12):6985–91.

4. Uzel BPC, Takano GHS, Chartuni JCN, Cesetti MV, Gavioli CFB, Lemes AM, et al. Intradermal injections with 0.5% minoxidil for the treatment of female androgenetic alopecia: A randomized, placebo-controlled trial. Dermatol Ther. 2021 Jan;34(1):e14622.

5. Yu CQ, Zhang H, Guo ME, Li XK, Chen HD, Li YH, et al. Combination therapy with topical minoxidil and nano-microneedle-assisted fibroblast growth factor for male androgenetic alopecia: a randomized controlled trial in Chinese patients. Chin Med J (Engl). 2020 Nov 5;134(7):851–3.

6. Ferrara F, Kakizaki P, de Brito FF, Contin LA, Machado CJ, Donati A. Efficacy of Minoxidil Combined With Photobiomodulation for the Treatment of Male Androgenetic Alopecia. A Double-Blind Half-Head Controlled Trial. Lasers Surg Med. 2021 Nov;53(9):1201–7.

7. Begum S, Lee MR, Gu LJ, Hossain MJ, Kim HK, Sung CK. Comparative hair restorer efficacy of medicinal herb on nude (Foxn1nu) mice. BioMed Res Int. 2014;2014:319795.

8. Wang Q, Wang Y, Pang S, Zhou J, Cai J, Shang J. Alcohol extract from Vernonia anthelmintica willd (L.) seed counteracts stress-induced murine hair follicle growth inhibition. BMC Complement Altern Med. 2019 Dec;19(1):372.

9. Kim BH, Lee MJ, Lee WY, Pyo J, Shin MS, Hwang GS, et al. Hair Growth Stimulation Effect of Centipeda minima Extract: Identification of Active Compounds and Anagen-Activating Signaling Pathways. Biomolecules. 2021 Jul 2;11(7):976.

10. Ruksiriwanich W, Khantham C, Muangsanguan A, Phimolsiripol Y, Barba FJ, Sringarm K, et al. Guava (Psidium guajava L.) Leaf Extract as Bioactive Substances for Anti-Androgen and Antioxidant Activities. Plants Basel Switz. 2022 Dec 14;11(24):3514.

11. Luo Z, Zhang X. Brassica oleracea extract, glucosinlates, and sulforaphane promote hair growth in vitro and ex vivo. J Cosmet Dermatol. 2022 Mar;21(3):1178–84.

12. Liu X, Ji T, Hu H, Lv X, Zhu G. The Hair Growth-Promoting Effect of Gardenia florida Fruit Extract and Its Molecular Regulation. Evid-Based Complement Altern Med ECAM. 2022;2022:8498974.

13. Zhang Y, Chen S, Qu F, Su G, Zhao Y. In vivo and in vitro evaluation of hair growth potential of Cacumen Platycladi, and GC-MS analysis of the active constituents of volatile oil. J Ethnopharmacol. 2019 Jun;238:111835.

14. Nanashima N, Horie K. Blackcurrant Extract with Phytoestrogen Activity Alleviates Hair Loss in Ovariectomized Rats. Mol Basel Switz. 2019 Apr 1;24(7):1272.

15. Im J, Hyun J, Kim SW, Bhang SH. Enhancing the Angiogenic and Proliferative Capacity of Dermal Fibroblasts with Mulberry (Morus alba. L) Root Extract. Tissue Eng Regen Med. 2022 Feb;19(1):49–57.

16. Lee TK, Kim B, Kim DW, Ahn JH, Sim H, Lee JC, et al. Effects of Decursin and Angelica gigas Nakai Root Extract on Hair Growth in Mouse Dorsal Skin via Regulating Inflammatory Cytokines. Mol Basel Switz. 2020 Aug 13;25(16):3697.
